# Diversity and distribution of *Oxytropis* DC. (Fabaceae) species in Asian Russia

**DOI:** 10.3897/BDJ.10.e78666

**Published:** 2022-01-20

**Authors:** Denis V. Sandanov, Anastasiia S. Dugarova, Elena P. Brianskaia, Inessa Yu. Selyutina, Natalia I. Makunina, Sergey V. Dudov, Victor V. Chepinoga, Zhiheng Wang

**Affiliations:** 1 Institute of General and Experimental Biology SB RAS, Ulan-Ude, Russia Institute of General and Experimental Biology SB RAS Ulan-Ude Russia; 2 Central Siberian Botanical Garden SB RAS, Novosibirsk, Russia Central Siberian Botanical Garden SB RAS Novosibirsk Russia; 3 Moscow State University, Moscow, Russia Moscow State University Moscow Russia; 4 Irkutsk State University, Irkutsk, Russia Irkutsk State University Irkutsk Russia; 5 Peking University, Beijing, China Peking University Beijing China

**Keywords:** dataset, occurrences, digitised printed maps, field observations, diversity patterns

## Abstract

**Background:**

The dataset providing information on the geographic distribution of *Oxytropis* species on the territory of Asian Russia is discussed. The data were extracted from different sources including prominent floras and check-lists, Red Data books, published research on congeneric species and authors’ field observations and mainly cover less-studied, remote regions of Russia. The dataset should be of value to applied, basic and theoretical plant biologists and ecologists interested in the *Oxytropis* species.

**New information:**

The dataset includes 5172 distribution records for 143 species and 15 subspecies of genus *Oxytropis* DC. (Fabaceae Lindl.) in Asian Russia. The dataset fills gaps in the distribution of locoweeds in the study area and contains precise coordinates for many of rare and endemic species.

## Introduction

*Oxytropis* DC. or Locoweeds is one of the richest genera in the flora of non-tropical Asia (Malyshev 2008). There are 378 species and subspecies in northern Eurasia (Yakovlev et al. 1996), 154 species and 24 subspecies in Siberia and the Russian Far East (Baikov 2012). However, according to the latest revision for the Asian part of Russia, the genus includes 142 species and 24 subspecies (Malyshev 2008).

Leonid Malyshev (Malyshev 2006) considered *Oxytropis* as a critical genus in plant biogeography because the areas with a high diversity of these species in north and central Asia differed considerably from each other by their quaternary history as well as their ecological and geographical specialisation. The highest diversity of locoweeds in Asian Russia is confined to the mountainous territories (Malyshev 2006, Malyshev 2008). The genus is characterised by a wide distribution of interspecific hybridisation and rapid adaptive radiation of many young species (Kholina et al. 2016).

The insufficient data on the distribution of *Oxytropis* species in Asian Russia (especially for scattered and endemic species) stimulated a surge of research interest in this genus. As a result, different studies dealing with various aspects of the ecology, distribution and taxonomic status of selected sections and model species have been published recently (Gureyeva and Bytotova 2001, Konichenko and Selyutina 2013, Kholina et al. 2018, Selyutina and Sandanov 2018).

These studies revealed an extensive array of new localities and expanded our knowledge about many rare and endemic *Oxytropis* species (Selyutina et al. 2010, Chimitov et al. 2017). Detailed studies of population biology features of selected endemic *Oxytropis* were also recently started by several authors (Selyuitina et al. 2018, Selyutina et al. 2019, Sandanov 2019a, Sandanov et al. 2020). Finally, adding molecular biology methods to researchers' toolkits made it possible to answer some other challenging questions in taxonomy, phylogenetics and phylogeography of the genus (Artyukova et al. 2004, Shahi Shavvon et al. 2017, Kholina et al. 2018, Kholina et al. 2021).

Analysing species distribution patterns and bioclimatic modelling usually requires large datasets (Araújo et al. 2019). Application of such methods helps to understand the present patterns of plant species distribution and their potential response to climate change and human impact and aids developing the conservation measures for endangered plants (Sandanov 2019b, Sandanov 2020). Thus, the presented dataset contains key information crucial for conducting different kinds of research, taking *Oxytropis* as a model object.

## General description

### Purpose

The aims of this study were: 1) to digitise species distribution maps of *Oxytropis* species in Asian Russia from available sources, 2) to summarise authors’ field data on locoweeds and 3) to merge all data to a single dataset.

## Project description

### Title

№ 121030900138-8 «Biota of terrestrial ecosystems of Baikal Region: composition, structure, eco-geographic patterns»

### Personnel

Denis Sandanov, Anastasia Dugarova, Elena Brianskaia

### Study area description

Baikal Region, Russia. Coordinates: 49.00 and 64.00 Latitude; 98.77 and 122.00 Longitude (WGS84).

### Design description

The project involves different disciplines: flora and plant taxonomy, plant biology and population ecology, the vegetation of Baikal Region, fauna and ecology of insects, ecology and geography of vertebrates.

### Funding

Russian Federal Budget

## Sampling methods

### Study extent

Asian Russia (or Asian part of Russia) is a vast territory that occupies 1/3 of Asia or about 13,100,000 km^2^ and stretches for more than 7000 km from east to west and almost 4500 km from the north to south (Xue et al. 2020).

### Sampling description

In total, we have digitised 124 distribution maps of *Oxytropis* species and subspecies (except for ecotypes) from several key floras and check-lists (Sandanov et al. 2021) (Table 1). The compendium “Arctic Flora of USSR” contains 45 distribution maps of studied species and subspecies (Yurtzev 1986). We recognised all seven subspecies of *O.middendorffii* in accordance with the 'GBIF backbone' and paper by Leonid Malyshev (Malyshev 2008). Original data for *O.middendorffii* s.l. are also presented in the dataset. We did not digitise maps from the “Flora of Siberia” which included 47 species and five subspecies of *Oxytropis* (Polozhij 1994), because this part of the data was previously published by Artemov and Egorova (Artemov and Egorova 2021). The compendium “Vascular Plants of Soviet Far East” includes 49 locoweed species (Pavlova 1989). Some of them are nowadays considered as subspecies, i.e. O.middendorffiisubsp.anadyrensis and O.middendorffiisubsp.trautvetteri. Occurrences from the “Flora of Central Siberia” (Peshkova 1979) were derived automatically using a special programme on Java and later verified manually (Chepinoga et al. 2017). The distribution of 30 *Oxytropis* species from the “Flora of Central Siberia” were adjusted to the grid system used in the compendium. The other source, the atlas “Endemic Alpine Plants of Northern Asia,” also contains information on the distribution of 13 locoweed species with 281 occurrences in Asian Russia. We have not considered this source, while it was recently published in a separate dataset (Brianskaia et al. 2021a, Brianskaia et al. 2021b).

The digitalisation was performed in QGIS 3.10 and QGIS 3.16 software through the georeferencing tool. We georeferenced the source raster distribution maps by snapping control points to the destination vector shapefile. We used the Natural Earth vector map at 1:10 m scale as the base map for georeferencing. Control points linked raster maps to the destination shapefile, which resulted in a transformation of the maps according to the spatial projection of destination features (WGS 1984). Subsequently, we digitised species distribution locations from each map. Coordinates of each location were calculated in an attribute table.

Denis Sandanov with Elena Brianskaia georeferenced distribution maps from “Arctic Flora of USSR” (Yurtzev 1986), Sergey Dudov – maps from “Vascular Plants of Soviet Far East” (Pavlova 1989). Victor Chepinoga provided coordinates for localities of *Oxytropis* from “Flora of Central Siberia” (Chepinoga et al. 2017) (Table 2).

Additionally, we georeferenced maps from Red Data Books published for different regions of Russia, i.e. Republic of Khakassia (Krasnoborov 2012), Republic of Buryatia (Pronin 2013), Altai Republic (Maneev 2017), Republic Sakha (Yakutia) (Danilova 2017), Zabaikalsky Krai (Polyakov 2017), Altai Krai (Shmakov and Silantyeva 2016) and Irkutsk Oblast (Trofimova 2020). In some cases, Red Data Books contain distribution data not available in other sources. Some additional information was derived from papers by Malyshev (Malyshev 2008) and by Pyak (Pyak 2014).

A considerable number of occurrences were extracted from authors’ original field data (relevés, field diaries etc.). The main contributors were Denis Sandanov (1332 occurrences), Natalia Makunina (704 occurrences) and Inessa Selyutina (408 occurrences). The dataset, presented in this paper, is the only source for the occurrence of some rare and locally distributed *Oxytropis* species in GBIF. For a few such species, we provide exact coordinates obtained from the field observations. The highest percentage of coordinates with high precision (more than 50%) is presented for 59 species (Table 3).

**Figure 1.**
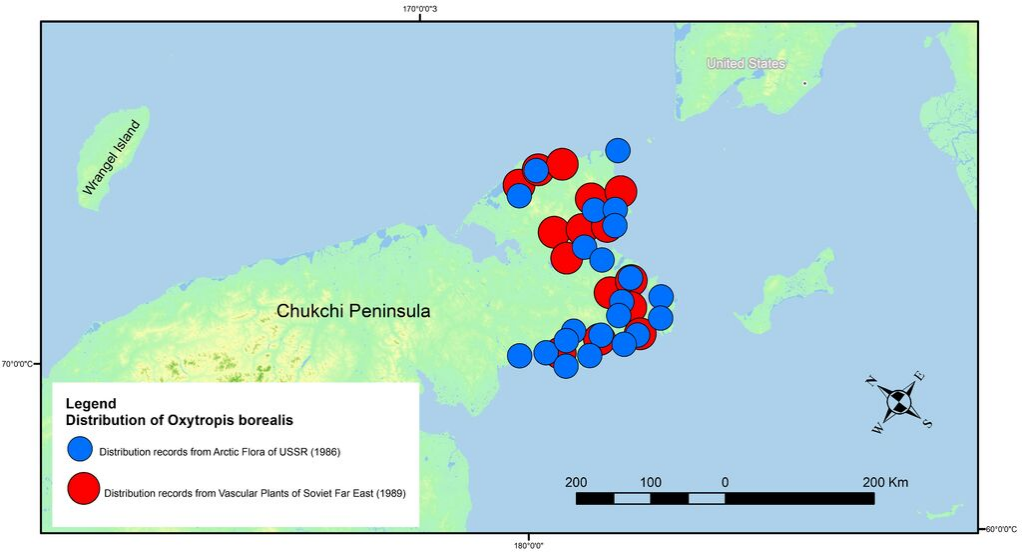
Distribution of *Oxytropisborealis* Note: Map is presented in Albers Equal Area Conic projection. Size of symbols is similar to symbols on distribution maps of digitised flora.

**Figure 2.**
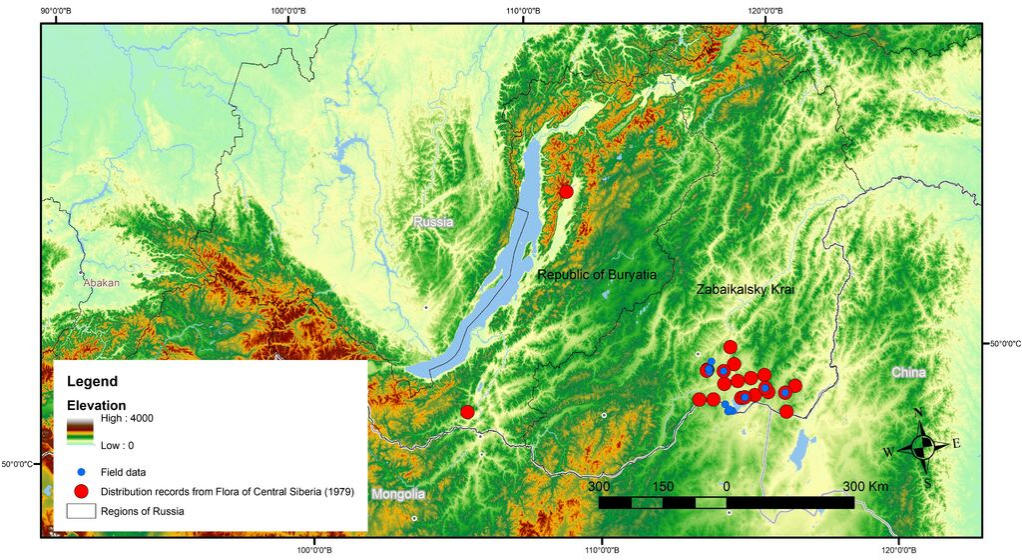
Distribution of *Oxytropisfiliformis* Note: Map is presented in Albers Equal Area Conic projection. Size of symbols is similar to symbols on distribution maps of digitised flora.

The majority of the data do not overlap because they are presented for different regions of Asian Russia. Data overlapping is observed for some arctic species with the distribution covering the north-eastern part of Asian Russia. The distribution map for *Oxytropisborealis* revealed overlapping of several distribution records (Fig. 1). Distribution data from “Arctic flora of USSR” provide more details and have higher resolution in comparison with species occurrences from “Vascular Plants of Soviet Far East”. Digitised distribution records from floras, in some cases, are complemented by field data which have better spatial resolution and can be used for large-scale mapping (Fig. 2). For example, the distribution of *Oxytropisfiliformis* has five overlapping field records with data from “Flora of Central Siberia”, but field data do not cover the whole distribution of studied species. Therefore, combining distribution data from different sources reveals more occurrences in various habitats which are helpful for understanding ecological features of species and future species distribution modelling.

### Quality control

Final examination of the digitised species distribution maps was performed in QGIS 3.10 and QGIS 3.16. For each species, we compared the output digitised occurrences with the original maps. Following Chapman and Wieczorek (Chapman and Wieczorek 2021), we applied three types of coordinate uncertainties. The first type includes coordinate uncertainty of species occurrence from the herbarium locality description. For such data, we established approximated 5 km as the coordinate uncertainty. The second type is the coordinate uncertainty of the drawn maps. Here, we accept a symbol diameter and map scale as a value of coordinate uncertainty at geocoding. Projections for the “Arctic Flora of USSR” (Yurtzev 1986) and “Vascular Plants of Soviet Far East” (Pavlova 1989) were recognised in QGIS. The original printed maps contained non-projective distortions; therefore, the initial rasters were transformed using the “polynomial 1” or “polynomial 2” transformation method and the "nearest neighbour" interpolation method. The largest possible source of error relates to the original mapping of points in the pre-digital era. To assess the impact of these uncertainties, we compared the position of points with known coordinates and points on maps, based on these samples. The spatial resolution of digitised maps was different: markers on the maps from the “Vascular Plants of Soviet Far East” have diameters from 33 to 45 km (mean = 39 km), “Flora of Siberia” – from 29 to 39 km (34 km), “Arctic Flora of USSR” – from 23 to 27 km (27 km). We used these mean parameters to estimate uncertainty for each digitised source. The third type is the coordinate uncertainty of the map digitalisation in QGIS. To test the coordinate uncertainty of such maps, three experts independently performed digitalisation on their computers for each type of map. As a result, the coordinate uncertainty was less than 5 km in all cases.

We calculated the final coordinate uncertainty by summarising all three types mentioned above and, for prominent flora, it is estimated as 49 km for the “Vascular Plants of Soviet Far East”, 44 km for “Flora of Siberia” and 37 km for “Arctic Flora of USSR”. Coordinate’s uncertainty for the grid maps of “Flora of Central Siberia” was considered as 18 km (Chepinoga et al. 2017). Occurrence data presented in the paper by Malyshev (Malyshev 2008) has lower precision and recognised uncertainty for them is equal to 30 km. The uncertainty of georeferenced data for Red Data Books, based on large-scale maps, was estimated as 5 km. The same accuracy was used for occurrences of *Oxytropissobolevskajae* (Pyak 2014).

### Step description

1. Digitising species distribution maps of *Oxytropis* species from prominent flora and check-lists of Asian Russia
2. Summarising field data with occurrences of locoweeds
3. Merging all data to a single dataset.

## Geographic coverage

### Description

**Figure 3.**
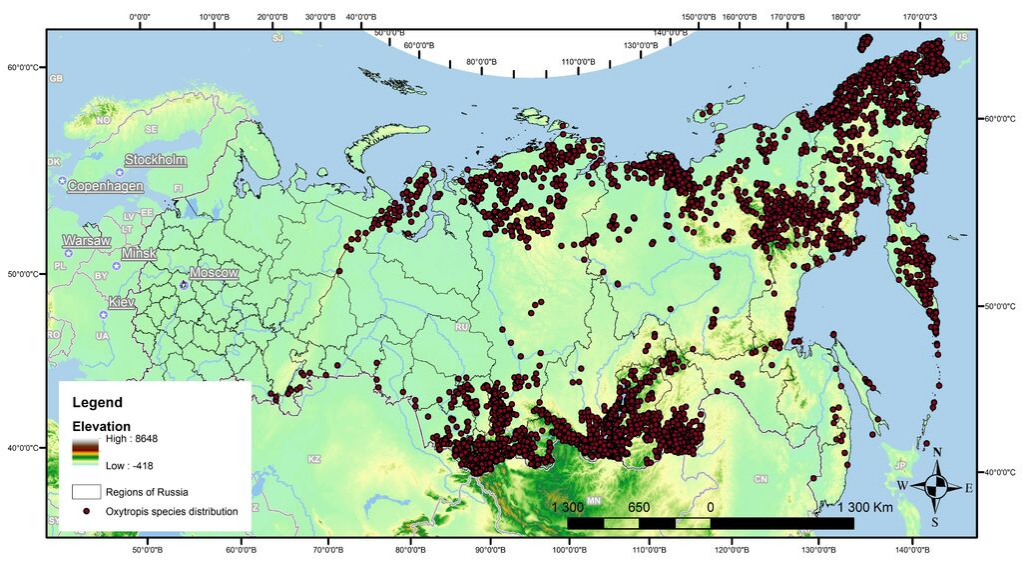
Distribution of *Oxytropis* species in Asian Russia. Note: Map is presented in Albers Equal Area Conic projection.

All *Oxytropis* species distribution records are located in Asian Russia. This is a vast area, stretching from the Ural Mountains in the west to the Pacific Ocean in the east; from the Arctic Ocean in the north to borders with Kazakhstan, Mongolia, China and northern Korea in the south. Analysis of diversity for all species presented in the study region has been done with adding the data from “Flora of Siberia” (Artemov and Egorova 2021). Very sparse distribution data were observed from the south-western part of Asian Russia, including Omsk Oblast, Novosibirsk Oblast, Tomsk Oblast, Tumen Oblast, Kurgan Oblast and Khanty-Mansi Autonomous Okrug (Fig. 3). A small part of occurrences was presented for the south part of Yamal-Nenets Autonomous Okrug, mid-latitudes of Krasnoyarsk Krai, the central and southern regions of Yakutia, Amur Oblast, Khabarovsk Krai and Primorsky Krai. Notably, the territory of the Sverdlovsk Oblast and Chelyabinsk Oblast (Central and Southern Ural Region) has not been included in the “Flora of Siberia” (Xue et al. 2020). That is the reason why the distribution data for the Ural Mts. are missing in this comprehensive compendium. Distribution maps for all analysed flora and check-lists are based on herbaria specimen. Thus, the published dataset on the distribution of *Oxytropis* species in Asian Russia reflects the absence of locoweeds and, in some cases, data from the region. The author’s field observations provided additional data from southern Siberia and northeast Asia. The main lack of information falls in the mid-latitudes of Asian Russia; the pattern is specific for vascular plants of northern Asia in general (Sandanov 2020). Recent activities in the “Flora of Russia” project, implemented on the iNaturalist platform (Seregin et al. 2020), involved many fine-scale distribution data for plant species, which can improve our general understanding of plant distribution and *Oxytropis* species, in particular.

**Figure 4.**
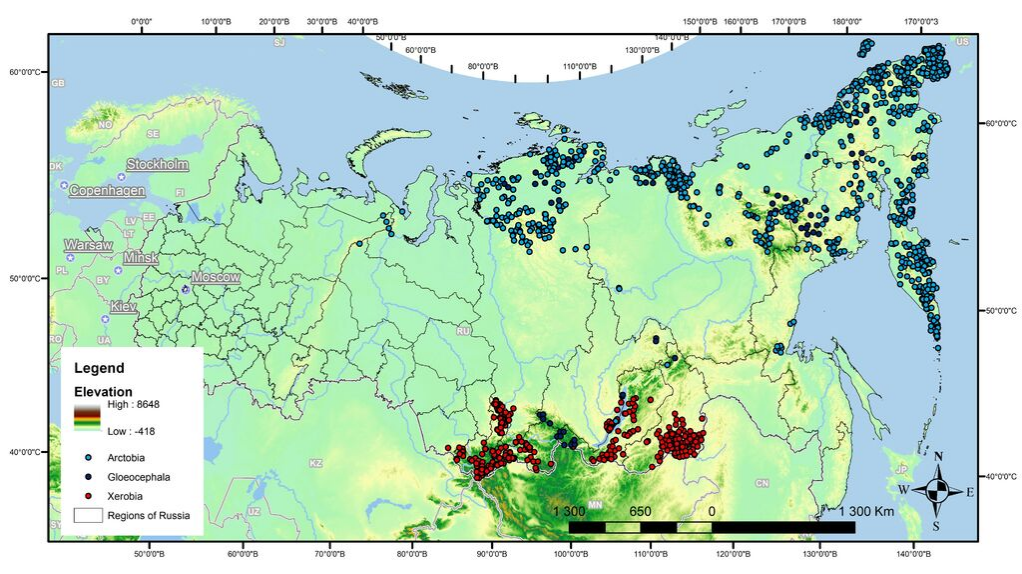
Distribution of species from sections *Arctobia*, *Gloeocephala* and *Xerobia*.

The distribution of locoweeds from different taxonomic sections showed a few interesting geographic patterns (Fig. 4). For example, section Xerobia is mainly distributed in central Asia, while sections *Arctobia* and *Gloeocephala* are most abundant in Asian Arctic. Some species from section Gloeocephala (e.g. *O.adenophylla*, *O.jurtzevii*) are distributed in the mountains of southern Siberia.

**Figure 5.**
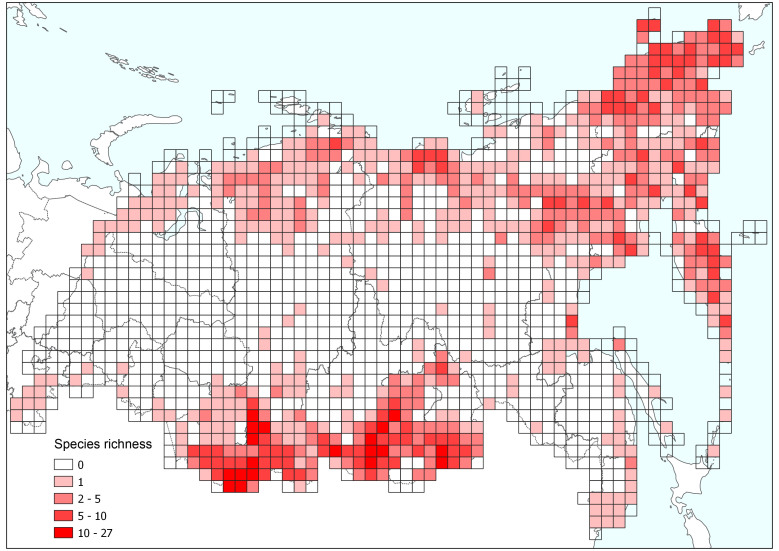
Patterns in species richness of genus *Oxytropis* in Asian Russia.

The distribution maps were transferred into gridded distributions with equal area projection (Albers cubic equal area projection) at a spatial resolution 100 × 100 km^2^ in QGIS to eliminate the potential influence of area on the estimation of species richness. The richness of *Oxytropis* species peaked in the mountainous regions of southern Siberia (Altai and Sayan Mts.) and north-eastern Asian Russia (Fig. 5). The topography of the latter region is complicated and includes a number of mountain ridges divided by plateaus and depressions. Higher diversity of *Oxytropis* in the territory of Baikal Siberia match with high-abundant floristic regions presented by Malyshev (Malyshev 2000).

### Coordinates

42.41 and 78.55 Latitude; 30.40 and 171.32 Longitude.

## Taxonomic coverage

### Description

The dataset covers infrageneric taxa of *Oxytropis* DC. (Fabaceae Lindl.) occurring in Asian Russia and includes 143 species and 15 subspecies according to 'GBIF backbone,' by the 'Species Matching Tool' (https://www.gbif.org/tools/species-lookup) (Table 3). Following Leonid Malyshev (Malyshev 2008), species with unclear taxonomic status were excluded from the dataset, i.e. *O.albiflora* Bunge, *O.bracteolata* Vass., *O.calva* Malyschev, *O.czerskii* Jurtzev, *O.dubia* Turcz., *O.fischeriana* Vass., *O.interposita* Sipl., *O.kateninii* Jurtzev, *O.kunashiriensis* Kitam., *O.malacophylla* Bunge, *O.protopopovii* Kom., *O.siegizmundii* N.S. Pavlova and *O.susumanica* Jurtzev. All of these species were observed in a few localities in the study region.

## Temporal coverage

### Notes

Dates of records range from 1946 to 2021. Distribution data for studied species in “Flora of Central Siberia” published in 1979, “Arctic Flora of USSR” in 1986 and “Vascular Plants of Soviet Far East” in 1989. Field observation data derived from 1991 till 2021.

## Usage licence

### Usage licence

Creative Commons Public Domain Waiver (CC-Zero)

### IP rights notes

This work is licensed under a Creative Commons Attribution (CC-BY) 4.0 Licence.

## Data resources

### Data package title

Occurrences of Oxytropis species on the territory of Asian Russia.

### Resource link

https://www.gbif.org/dataset/868e92bb-d93e-4fc9-886f-81149ec92ec2

### Alternative identifiers

http://gbif.ru:8080/ipt/resource?r=oxytropis_asian_russia

### Number of data sets

1

### Data set 1.

#### Data set name

Occurrences of Oxytropis species on the territory of Asian Russia.

#### Data format

Darwin Core Archive format.

#### Number of columns

27

#### Character set

UTF-8

#### Download URL

https://doi.org/10.15468/3vcw7y

#### Data format version

1.6

#### Description

The dataset providing information on the geographic distribution of *Oxytropis* species on the territory of Asian Russia is discussed. Different sources (prominent flora and compendia, modern papers, Red Data Books and field data) have been used to describe diversity and distribution of the one of the richest genera in northern and central Asia. Presented species distribution data cover purely studied regions of Russia and reveal different geographic patterns on species and supraspecific levels of organisation. The presented dataset will be helpful in understanding ecological features and main determinants limiting distribution of *Oxytropis* pecies.

**Data set 1.** 

Column label	Column description
occurrenceID	An identifier for the record, unique within this dataset. An abbreviation in the identifier' number (OxAsRus).
basisOfRecord	The specific nature of the data record in standard label of one of the Darwin Core classes: MachineObservation/HumanObservation.
eventDate	The date-time or interval during which an Event occurred. For occurrences, this is the date-time when the event was recorded. Not suitable for a time in a geological context.
scientificName	The full scientific name of the species according to GBIF taxonomy
genus	The full scientific name of the genus in which the taxon is classified.
specificEpithet	The name of the species epithet according to GBIF taxonomy.
infraspecificEpithet	The name of the lowest or terminal infraspecific epithet according to GBIF taxonomy.
associatedReferences	A list (concatenated and separated) of identifiers (publication, bibliographic reference, global unique identifier, URI) of literature associated with the Occurrence.
kingdom	The full scientific name of the kingdom in which the taxon is classified.
phylum	The full scientific name of the phylum or division in which the taxon is classified.
class	The full scientific name of the class in which the taxon is classified.
order	The full scientific name of the order in which the taxon is classified.
family	The full scientific name of the family in which the taxon is classified.
taxonRank	The taxonomic rank of the most specific name in the scientificName.
decimalLatitude	The geographic latitude (in decimal degrees, using the spatial reference system given in geodeticDatum) of the geographic centre of a Location.
decimalLongitude	The geographic longitude (in decimal degrees, using the spatial reference system given in geodeticDatum) of the geographic centre of a Location.
geodeticDatum	The ellipsoid, geodetic datum or spatial reference system (SRS) upon which the geographic coordinates given in decimalLatitude and decimalLongitude are based.
coordinateUncertaintyInMetres	The horizontal distance (in metres) from the given decimalLatitude and decimalLongitude describing the smallest circle containing the whole of the Location.
verbatimCoordinateSystem	The coordinate format for the verbatimLatitude and verbatimLongitude or the verbatimCoordinates of the Location.
georeferencedBy	A list of persons who determined the georeference (spatial representation) for the Location.
recordedBy	A list of persons who collected field data.
higherGeography	A list of geographic names less specific than the information captured in the locality term.
continent	The name of the continent in which the Location occurs.
countryCode	The standard code for the country in which the Location occurs.
type	The nature or genre of the resource.
language	A language of the resource.
licence	A legal document giving official permission to do something with the resource.

## Figures and Tables

**Table 1.** The list of *Oxytropis* species with georeferenced distribution maps. Column “*Oxytropis* species” contains original species name given in presented source.

Source	*Oxytropis* species
Yurtsev BA (1986) *Oxytropis* DC. // Arctic Flora of USSR. Nauka, Leningrad. Vol. 9, part 2. P. 61–146. [In Russian].	O.adamsianasubsp.adamsiana, O.adamsianasubsp.janensis, *O.ajanensis*, O.arcticasubsp.taimyrensis, *O.beringensis*, *O.borealis*, *O.bryophila*, *O.czukotica*, *O.deflexa*, O.deflexasubsp.dezhnevii, *O.evenorum*, *O.exserta*, *O.gorodkovii*, *O.inopinata*, *O.kamtschatica*, *O.katangensis*, O.leucanthasubsp.subarctica, O.leucanthasubsp.tschukotcensis, *O.maydelliana*, *O.mertensiana*, O.middendorffiisubsp.anadyrensis, O.middendorffiisubsp.coerulescens, O.middendorffiisubsp.jarovoji, O.middendorffiisubsp.orulganica, O.middendorffiisubsp.schmidtii, O.middendorffiisubsp.submiddendorfii, O.middendorffiisubsp.trautvetteri, *O.nigrescens*, *O.ochotensis*, *O.putoranica*, *O.revoluta*, *O.schmorgunoviae*, *O.semiglobosa*, *O.sordida*, O.sordidasubsp.arctolenensis, O.sordidasubsp.schamurinii, O.sordidasubsp.sordida, *O.sublongipes*, *O.sverdrupii*, *O.tichomirovii*, *O.uschakovii*, O.vassilczenkoisubsp.substepposa, O.vassilczenkoisubsp.vassilczenkoi, *O.vasskovskyi*, *O.wrangelii*
Pavlova NS (1989) Fabaceae // Vascular Plants of Soviet Far East. Nauka, Leningrad. Vol.4. P. 191–339. [In Russian].	*O.ajanensis*, *O.anadyrenis*, *O.austrosachalinensis*, *O.beringensis*, *O.borealis*, *O.bryophila*, *O.calcareorum*, *O.caespitosa*, *O.chankaensis*, *O.charkeviczii*, *O.czukotica*, *O.darpirensis*, *O.deflexa*, *O.erecta*, *O.evenorum*, *O.exserta*, *O.gorodkovii*, *O.helenae*, *O.hidakamontana*, *O.itoana*, *O.leucantha*, *O.litoralis*, *O.kamtschatica*, *O.kusnetzovii*, *O.mandshurica*, *O.maydelliana*, *O.mertensiana*, *O.middendorffii*, *O.muricata*, *O.ochotensis*, *O.pumilio*, *O.retusa*, *O.revoluta*, *O.rubricaudex*, *O.ruthenica*, *O.sachalinensis*, *O.scheludjakovae*, *O.schmorgunoviae*, *O.sordida*, *O.strobilacea*, *O.sverdrupii*, *O.tilingii*, *O.todomoshiriensis*, *O.trautvetteri*, *O.uschakovii*, *O.vassilczenkoi*, *O.vassilievii*, *O.vasskovskyi*, *O.wrangelii*
Peshkova GA (1979) *Fabaceae* or *Leguminosae* // Flora of the Central Siberia. Nauka, Novosibirsk. Vol. 2. P. 585–639. [In Russian].	*O.adenophylla*, *O.alpicola*, *O.altaica*, *O.baicalia*, *O.bargusinensis*, *O.coerulea*, *O.filiformis*, *O.glandulosa*, *O.grandiflora*, *O.heterotricha*, *O.jurtzevii*, *O.kodarensis*, *O.kusnetzovii*, *O.leptophylla*, *O.leucotricha*, *O.longirostra*, *O.mixotriche*, *O.muricata*, *O.myriophylla*, *O.oxyphylla*, *O.oxyphylloides*, *O.pilosa*, *O.popoviana*, *O.prostrata*, *O.squamulosa*, *O.stukovii*, *O.subnutans*, *O.sylvatica*, *O .turczaninovii*, *O.varlakovii*

**Table 2.** The list of persons who georeferenced distribution maps from different sources

Source	Georeferenced by	Num. of records
Yurtsev BA (1986) *Oxytropis* DC. // Arctic Flora of USSR. Nauka, Leningrad. Vol. 9, part 2. P. 61–146. [In Russian].	Denis Sandanov & Elena Brianskaia	1435
Pavlova NS (1989) Fabaceae // Vascular Plants of Soviet Far East. Nauka, Leningrad. Vol.4. P. 191–339. [In Russian].	Sergey Dudov	752
Peshkova GA (1979) *Fabaceae* or *Leguminosae* // Flora of Central Siberia. Nauka, Novosibirsk. Vol. 2. P. 585–639. [In Russian].	Viktor Chepinoga	447
Malyshev LI (2008) Diversity of the genus *Oxytropis* in Asian Russia // Turczaninowia. Vol. 11, no. 4, P. 5–141. [In Russian].	Denis Sandanov	45
Pyak AI (2014) *Oxytropissobolevskajae* sp. nov. (Fabaceae: Papilionoideae, Galegeae) from Tuva Republic (south Siberia, Russia) // Nordic Journal of Botany. Vol. 32. P. 139–142.	Anastasiia Dugarova	9
Red Data Book of Irkutsk Oblast (2020) Ulan-Ude. 552 p. [In Russian].	Denis Sandanov	19
Red Data Book of Altai Krai. Rare and deserving protection species of plants (2020) Barnaul. Vol. 1. 262 p. [In Russian].	Denis Sandanov	9
Red Data Book of the Altai Republic (2017) Gorno-Altaisk, 2017. 267 p. [In Russian].	Denis Sandanov	5
Red Data Book of the Republic Sakha (Yakutia) (2017) Rare and deserving protection species of plants and fungi. Moscow. Vol. 1. 412 p. [In Russian].	Denis Sandanov	4
Red Data Book of Republic of Buryatia: Rare and endangered species of animals, plants and fungi (2013) Ulan-Ude. 688 p. [In Russian].	Denis Sandanov	1
Red Data Book of the Republic Khakassia (2012) Krasnoyarsk-Abakan. 288 p. [In Russian].	Denis Sandanov	1
Red Data Book of Zabaikalsky Krai. Plants (2017) Novosibirsk. 384 p. [In Russian].	Denis Sandanov	1
Field observation data	Denis Sandanov, Natalia Makunina & Inessa Selyutina	2444
Total		5172

**Table 3.** Number of records for each *Oxytropis* species in the dataset, percentage of records with high coordinate precision. Species are listed in alphabetic order.

scientificName	Num. of records	Percent of fine-scale records	Scientific Name	Num. of records	Percent of fine-scale records
*Oxytropisacanthacea* Jurtzev	2	-	*Oxytropislongirostra* DC.	14	-
*Oxytropisaciphylla* Ledeb.	10	100	*Oxytropismacrosema* Bunge	17	100
*Oxytropisadamsiana* (Trautv.) Jurtzev	79	6.3	*Oxytropismandshurica* Bunge	24	-
Oxytropisadamsianasubsp.janensis Jurtzev	23	-	*Oxytropismartjanovii* Krylov	3	100
*Oxytropisadenophylla* Popov	5	-	*Oxytropismaydelliana* Trautv.	170	-
*Oxytropisajanensis* (Regel & Tiling) Bunge	4	25	*Oxytropismertensiana* Turcz.	147	-
*Oxytropisalpestris* Schischk.	4	100	*Oxytropismicrophylla* (Pall.) DC.	42	100
*Oxytropisalpicola* Turcz.	14	-	*Oxytropismiddendorffii* Trautv.	41	-
*Oxytropisalpina* Bunge	13	100	Oxytropismiddendorffiisubsp.albida Jurtzev	1	-
*Oxytropisaltaica* (Pall.) Pers.	18	11.1	Oxytropismiddendorffiisubsp.anadyrensis (Vassilcz.) Jurtzev	35	-
*Oxytropisammophila* Turcz.	1	100	Oxytropismiddendorffiisubsp.coerulescens Jurtzev & V.V.Petrovsky	12	-
*Oxytropisampullata* (Pall.) Pers.	4	100	Oxytropismiddendorffiisubsp.jarovoji Jurtzev	14	-
Oxytropisarcticasubsp.taimyrensis Jurtzev	55	-	Oxytropismiddendorffiisubsp.orulganica Jurtzev	15	-
*Oxytropisargentata* (Pall.) Pers.	6	83.3	Oxytropismiddendorffiisubsp.submiddendorffii Jurtzev	4	-
Oxytropisargentatasubsp.brevidentata Polozhij	1	-	Oxytropismiddendorffiisubsp.trautvetteri (Meinsh.) Jurtzev	15	-
*Oxytropisaustrosachalinensis* Vassilcz. ex N.S.Pavlova	1	-	*Oxytropismixotriche* Bunge	17	-
*Oxytropisbaicalia* (Pall.) Pers.	13	-	*Oxytropismuricata* (Pall.) DC.	52	75
*Oxytropisbargusinensis* Peschkova	101	89.1	*Oxytropismyriophylla* (Pall.) DC.	226	81.9
*Oxytropisberingensis* Jurtzev	2	-	*Oxytropisnigrescens* (Pall.) Fisch. ex DC.	137	-
*Oxytropisborealis* DC.	37	-	*Oxytropisnitens* Turcz.	24	100
*Oxytropisborissoviae* Polozhij	5	100	*Oxytropisnivea* Bunge	14	100
*Oxytropisbracteata* Basil.	1	100	*Oxytropisnuda* Basil.	67	100
*Oxytropisbryophila* (Greene) Jurtzev	3	-	*Oxytropisochotensis* Basil.	68	-
*Oxytropiscaespitosa* (Pall.) Pers.	83	98.8	*Oxytropisoligantha* Bunge	11	100
*Oxytropiscalcareorum* N.S.Pavlova	1	-	*Oxytropisoxyphylla* (Pall.) DC.	360	87.5
*Oxytropiscampanulata* Vassilcz.	104	100	*Oxytropisoxyphylloides* Popov	9	-
*Oxytropischakassiensis* Polozhij	16	100	*Oxytropispeschkovae* Popov	19	100
*Oxytropischankaensis* Jurtzev	11	-	*Oxytropispilosa* (L.) DC.	94	77.6
*Oxytropischarkeviczii* Vyschin	8	-	*Oxytropispolyphylla* Ledeb.	2	-
*Oxytropiscoerulea* (Pall.) DC.	52	19.2	*Oxytropispopoviana* Peschkova	12	58.3
*Oxytropisconfusa* Bunge	2	-	*Oxytropisprostrata* (Pall.) DC.	8	50
*Oxytropisczekanowskii* Jurtzev	3	-	*Oxytropispumila* Fisch. ex DC.	10	100
*Oxytropisczukotica* Jurtzev	192	2.6	*Oxytropispumilio* (Pall.) Ledeb.	28	-
*Oxytropisdarpirensis* Jurtzev & A.P.Khokhr.	1	-	*Oxytropisputoranica* M.M.Ivanova	4	-
*Oxytropisdeflexa* (Pall.) DC.	81	22.2	*Oxytropisrecognita* Bunge	17	100
Oxytropisdeflexasubsp.dezhnevii (Jurtzev) Jurtzev	2	-	*Oxytropisretusa* Matsum.	7	-
*Oxytropisdorogostajskyi* Kuzen.	2	-	*Oxytropisreverdattoi* Jurtzev	15	100
*Oxytropiserecta* Kom.	27	-	*Oxytropisrevoluta* Ledeb.	97	-
*Oxytropiseriocarpa* Bunge	65	100	*Oxytropisrubricaudex* Hulten	1	-
*Oxytropisevenorum* Jurtzev & A.P.Khokhr.	110	7.3	*Oxytropisruthenica* Vassilcz.	8	-
*Oxytropisexserta* Jurtzev	83	1.2	*Oxytropissachalinensis* Miyabe & Tatew.	5	-
*Oxytropisfiliformis* DC.	61	65.6	*Oxytropissaposhnikovii* Krylov	3	100
*Oxytropisfloribunda* (Pall.) DC.	11	2	*Oxytropisscheludjakovae* Karav. & Jurtzev	6	-
*Oxytropisgebleri* Fisch. ex Bunge	3	18.2	*Oxytropisschmorgunoviae* Jurtzev	14	-
*Oxytropisglabra* DC.	6	100	*Oxytropisselengensis* Bunge	12	100
*Oxytropisglandulosa* Turcz.	29	89.7	*Oxytropissemiglobosa* Jurtzev	42	-
*Oxytropisgorodkovii* Jurtzev	37	-	*Oxytropissetosa* (Pall.) DC.	25	100
*Oxytropisgrandiflora* DC.	114	82.5	*Oxytropissobolevskajae* Pjak	9	-
*Oxytropishailarensis* Kitag.	1	-	*Oxytropissongorica* (Pall.) DC.	2	100
*Oxytropishelenae* N.S.Pavlova	1	-	*Oxytropissordida* (Willd.) Pers.	60	-
*Oxytropisheterotricha* Turcz.	5	-	Oxytropissordidasubsp.arctolenensis Jurtzev	8	-
*Oxytropishidaka-montana* Miyabe & Tatew.	1	-	*Oxytropisspicata* (Pall.) O. et B. Fedtsch.	2	100
*Oxytropisinaria* (Pall.) DC.	3	100	*Oxytropissquammulosa* DC.	12	58.3
*Oxytropisincludens* Basil.	48	100	*Oxytropisstenofoliola* Polozhij	5	100
*Oxytropisinopinata* Jurtzev	4	-	*Oxytropisstenophylla* Bunge	29	100
*Oxytropisintermedia* Bunge	111	100	*Oxytropisstrobilacea* Bunge	195	96.9
*Oxytropisircutensis* Popov	3	-	*Oxytropisstukovii* Palib.	8	62.5
*Oxytropisitoana* Tatew.	1	-	*Oxytropissublongipes* Jurtzev	6	-
*Oxytropisjurtzevii* Malyschev	12	-	*Oxytropissubnutans* (Jurtzev) Jurtzev	11	-
*Oxytropiskamtschatica* Hulten	53	-	*Oxytropissuprajenissejensis* Kuvaev & Sonnikova	1	-
*Oxytropiskaravaevii* Jurtzev	1	-	*Oxytropissverdrupii* Lynge	5	-
*Oxytropiskaspensis* Krasnob. & Pshenich.	1	-	*Oxytropissylvatica* (Pall.) DC.	33	3
*Oxytropiskatangensis* Basil.	4	-	*Oxytropisteres* DC.	3	100
*Oxytropiskodarensis* Jurtzev & Malyschev	8	-	*Oxytropistichomirovii* Jurtzev	8	-
*Oxytropiskomarovii* Vassilcz.	1	-	*Oxytropistilingii* Bunge	8	-
*Oxytropiskomei* Saposhn.	1	-	*Oxytropistodomoshiriensis* Miyabe & T.Miyake	1	-
*Oxytropiskossinskyi* B. Fedtsch. & Basil.	7	100	*Oxytropistompudae* Popov	7	14.3
*Oxytropiskusnetzovii* Krylov & Steinb.	30	-	*Oxytropistragacanthoides* Fisch. ex DC.	22	100
*Oxytropisladyginii* Krylov	9	44.4	*Oxytropistrichophysa* Bunge	11	100
*Oxytropislanata* (Pall.) DC.	212	99.5	*Oxytropistriphylla* (Pall.) DC.	90	98.9
*Oxytropislasiopoda* Bunge	2	-	*Oxytropistschujae* Bunge	6	83.3
*Oxytropislanuginosa* Kom.	1	100	*Oxytropisturczaninovii* Jurtzev	164	81.7
*Oxytropislapponica* (Wahlenb.) J. Gay	5	100	*Oxytropisuschakovii* Jurtzev	11	-
*Oxytropisleptophylla* (Pall.) DC.	27	66.7	*Oxytropisvarlakovii* Serg.	8	75
*Oxytropisleucantha* (Pall.) Pers.	35	-	*Oxytropisvassilczenkoi* Jurtzev	162	-
Oxytropisleucanthasubsp.subarctica Jurtzev	26	-	Oxytropisvassilczenkoisubsp.substepposa Jurtzev	42	-
Oxytropisleucanthasubsp.tschukotcensis Jurtzev	72	-	*Oxytropisvassilievii* Jurtzev	2	-
*Oxytropisleucotricha* Turcz.	9	-	*Oxytropisvasskovskyi* Jurtzev	51	7.8
*Oxytropislitoralis* Kom.	9	-	*Oxytropiswrangelii* Jurtzev	13	-
